# What services are available for culturally and linguistically diverse (CALD) patients in the cancer survivorship setting? An Australian study

**DOI:** 10.1007/s00520-025-09348-2

**Published:** 2025-03-21

**Authors:** Lawrence Kasherman, Isaac Yeboah Addo, Sim Yee Cindy Tan, Ashanya Malalasekera, Joanne Shaw, Janette Vardy

**Affiliations:** 1 Concord Clinical School, Faculty of Medicine and Health, University of Sydney, Concord, Sydney, New South Wales 2138 Australia; 2 Department of Medical Oncology, Illawarra Cancer Care Centre, Wollongong, New South Wales Australia; 3 Sydney Cancer Survivorship Centre, Concord Cancer Centre, Concord Hospital, Concord, New South Wales Australia; 4 General Practice Clinical School, University of Sydney, Sydney, New South Wales Australia; 5 Psycho-Oncology Co-Operative Research Group (PoCoG), School of Psychology, University of Sydney, Sydney, New South Wales Australia

**Keywords:** Culturally and linguistically diverse, Cancer survivorship, Health service delivery, Qualitative interviews

## Abstract

**Purpose:**

People of Culturally and Linguistically Diverse (CALD) backgrounds face disparities in cancer care. This study aimed to explore CALD-specific Cancer Survivorship (CS) resources and supports in Australian oncology centres.

**Methods:**

This was an interview-based, qualitative study. Oncology professionals were interviewed using a questionnaire exploring demographics, available resources and referral patterns, and factors influencing CALD CS care. Purposive sampling was used to ensure representation across states and remoteness areas. Contextual survey data were analysed with descriptive statistics, and interviews were recorded and transcribed for thematic analysis.

**Results:**

Twenty-two interviews from 15 institutions across 6 Australian states were conducted from May to August 2023. Six (40%) centres reported seeing > 25% CALD patients. Six (40%) centres reported having dedicated CS services dichotomised into *clinic-based* or *needs-based* services. Ten (67%) centres reported having CALD-specific resources/supports for oncology patients, and three (20%) had CS-specific services. Four themes were identified: patient-clinician interface; in-language resources with a focus on cultural relevance; structural and logistical considerations, particularly interpreter services, workflow management and models of care; and education and collaboration between healthcare professionals and survivors, carers and community leaders.

**Conclusions:**

Cancer survivors from CALD backgrounds face unique challenges in receiving optimal care, with limited availability of CALD-specific resources in Australian cancer centres. Future work should utilise a tailored and collaborative approach to optimise cultural relevance and service engagement.

**Supplementary Information:**

The online version contains supplementary material available at 10.1007/s00520-025-09348-2.

## Introduction

In 2021, census data determined nearly 30% of Australians were born overseas, and over 25% reported speaking a language other than English at home [1]. The Australian Bureau of Statistics defines Culturally and Linguistically Diverse (CALD) people as those born overseas in countries other than those classified as ‘main English-speaking countries’ [2]. It is increasingly recognised that CALD communities face challenges with access to and delivery of optimal healthcare, and lead to inferior health outcomes. The impact of these disparities persists throughout the spectrum of cancer care, including diagnosis, treatment, survivorship and palliative care [3–6].

Cancer survivorship (CS) is a core component of oncology care delivery [7]. However, delivery of CS care varies by region and there is no current standardised model of care, although guidelines such as the Clinical Oncology Society of Australia (COSA) Model of Survivorship Care define the critical components [8]. Survivorship care delivery may be even more complex in CALD groups, with cultural sensitivity, community engagement and stakeholder involvement crucial in service and resource development, and their dissemination to optimise reach [9].

In recognising the growing CALD populations with cancer diagnoses [10], community support groups focused on specific cultural or language groups have been established in Australia. Prominent oncology stakeholder groups in Australia have developed translated patient resources, and have partnered with community support groups and area health services to develop culturally sensitive resources [11–13]. These groups endorse and support implementation studies focused on CALD communities across the cancer spectrum, which is particularly important given lower research participation rates in these populations. [14, 15]. However, projects specifically targeting CALD cancer survivors remain limited.

Our research group published a scoping review to explore existing literature around CALD-specific CS interventions and their impact [16]. Most studies focused on one specific CALD group and were delivered in-language, and most were focused on breast cancer-affected populations based in the United States. We concluded CS programs or interventions were associated with positive outcomes in CALD populations, although findings were difficult to generalise across different populations of cancer survivors. This study aimed to build upon this, exploring healthcare professionals’ identification of existing resources and support pathways for cancer survivors from CALD backgrounds in Australian cancer centres, and described potential CALD-specific factors that impact survivorship care delivery.

## Methods

### Methodological orientation and theory

This was a qualitative, interview-based scoping study. The Consolidated Criteria for Reporting Qualitative Research (COREQ) checklist [17] was used to guide reporting (Appendix 1). For the purposes of this study, we defined CALD [18] populations as those from non-English speaking backgrounds or people born outside of Australia whose first language is not English. This definition does not encompass Indigenous or First Nations populations as they are not migrant populations, and the historical contexts of invasion and dispossession have given way to unique socio-political and cultural issues impacting healthcare. Our definition of a cancer survivor was a person diagnosed with cancer in adulthood, who: had completed curative-intent cancer treatment including chemotherapy, radiation and/or targeted therapies, and were on surveillance alone; or those who had received, or were currently receiving, maintenance anti-cancer therapy for metastatic disease, and deemed to have stable disease or no evidence of active disease. A Cancer Survivorship (CS) service was defined as a healthcare service, usually multidisciplinary, aiming to provide complementary, supportive care for cancer survivors. Questionnaires were initially pilot tested for feasibility with five HCPs with involvement in CS.

### Participant selection

Eligible participants were oncology healthcare professionals (HCPs) who regularly treat patients diagnosed with adult-onset (≥ 18 years old) cancers. Interviewees could include directors of cancer services, medical or radiation oncologists, haematologists, nurses, allied health practitioners, practice/clinical managers who have experience or interest in CS.

Suitable centres were identified by searching Commonwealth Declared hospitals listed on the Australian Government Department of Health website [19]. Hospital websites were screened for availability of cancer services and email contact was made with heads of departments. A Participant Information Sheet was attached to the initial email, and link to a secure, web-based Research Electronic Data Capture (REDCap) e-Consent form and short survey.

Purposive sampling was used, aiming for national representation of each state and territory, and metropolitan, regional and rural centres using the Australian Statistical Geography Standard–Remoteness Areas [20, 21] (ASGS-RA), and between public and private sectors. Health districts with higher CALD populations were identified and approached [10]. Estimated sample size was 20 sites, with data assessed in real-time for theoretical saturation, with three further centres interviewed once saturation was identified to confirm no new information.

### Survey

Following e-Consent and prior to interviews, participants were requested to complete an initial REDCap questionnaire for participant demographic information, including their role, institution, postcode, length of work experience, percentage of CALD patients and most common CALD groups seen at their institution. Additional questions about participants’ country of birth and languages spoken at work were optional.

### Participant interviews

Interviews were conducted via Zoom with audio recording, exploring domains including survivorship services at the participants’ workplaces, referral patterns, patient demographics, services for CALD populations, and perceived factors impacting uptake of survivorship resources and services by those from CALD backgrounds. No non-participants were present during the interviews. Participants had the right to withdraw from the study at any time, including those who had completed the surveys but had not yet interviewed. Repeat interviews were not conducted, and transcripts were not returned to participants for comment.

### Data analysis

Quantitative data was summarized descriptively, with categorical data as percentages and ranges, and continuous data as means and ranges. As this study was applied research rather than hypothesis generating, the authors utilised reflexive thematic analysis approach outlined by Braun and Clarke [22]. Qualitative data was coded using NVivo 14 [23] by two authors (LK and IYA). Three transcripts were initially coded to ensure inter-reliability, and the initial codebook was reviewed by the research team to ensure consensus. LK and IYA met regularly to ensure ongoing agreement, and emergent themes were discussed within the research team.

### Research team and reflexivity

Interviews were conducted by LK, a male Medical Oncologist from CALD background. To ensure interpretation rigor, the research team employed techniques such as double coding, iterative nature of analysis, and regular discussions with the research team.

## Results

Interviews were conducted from May to August 2023 with 22 participants from 15 centres. Ten additional participants were contacted; 7 heads of department did not respond after two emails, and 3 interested participants who initially responded did not complete e-Consent. Mean interview length was 25 min.

### Participant and institutional characteristics

Participant demographic, clinical practice and institutional information is summarised in Table 1. Of 15 centres represented, most were public (11/15; 73%), metropolitan (12/15; 80%) centres from New South Wales (7/15; 46%). There was multi-disciplinary representation amongst interviewees, with Medical Oncologists comprising 50% (11/22). Most (13/22; 59%) had worked in oncology for more than 15 years.

**Table 1:** Demographic information of centres represented (22 interviewees across 15 centres)

Variables	N (%)
State	
New South Wales	7 (46)
Victoria	2 (13)
Queensland	3 (20)
Northern Territory	1 (7)
South Australia	1 (7)
Western Australia	1 (7)
2021 ASGS-RA/ARIA index	
1 (Major Cities)	12 (80)
2 (Inner Regional)	1 (7)
3 (Outer Regional)	2 (13)
4 (Remote)	0 (0)
5 (Very Remote)	0 (0)
Type of centre	
Public	11 (73)
Private	1 (7)
Mixed	3 (20)
Estimated % of CALD patients seen at their facility	
< 25%	9 (60)
26–50%	5 (33)
51–75%	1 (7)
76–100%	0 (0)
Country of birth by interviewee	
Australia	11 (50)
Europe	4 (18)
Asia	4 (18)
N/A	3 (14)
Language spoken other than English by interviewee	
Yes	7 (32)
No	14 (64)
N/A	1 (4)
Interviewee role at their current institution *	
Head of cancer services	2
Medical oncologist	11
Radiation oncologist	1
CNC/NP	4
Clinical psychologist	1
Other • General practitioner • Innovations manager • Health literacy and inclusion manager • Social worker	4
Duration of employment at institution by interviewee	
< 12 months	2 (9)
1–5 years	9 (41)
6–10 years	6 (27)
> 15 years	5 (23)
Duration of employment within cancer services by interviewee	
< 12 months	0 (0)
1–5 years	4 (18)
6–10 years	5 (23)
> 15 years	13 (59)

Abbreviations: *ASGS-RA*  Australian Statistical Geography Standard – Remoteness Areas; *ARIA*  Accessibility/Remoteness Index of Australia; *CALD*  Culturally and Linguistically Diverse; *N/A*  not available; *CNC*  clinical nurse consultant; *NP*  nurse practitioner

* indicates more than one response could be selected

In mapping most common CALD groups by institution, over 20 groups were identified including Chinese, Arabic, Greek, Italian and Vietnamese, and 40% (6/15) reported CALD groups comprised over 25% of patients seen at their facilities. Of the 19 participants who provided a response to questions about their country of birth and languages spoken, 42% (8/19) were born outside of Australia and 37% (7/19) spoke a language other than English fluently, including four participants who spoke their nominated language within a workplace setting.

Service characteristics are summarised in Table 2. Six (40%) centres reported having dedicated CS services, dichotomised into: *clinic-based*, a dedicated survivorship clinic where services or referrals are facilitated; or *needs-based*, services that could be accessed upon request, but not necessarily CS-specific. In terms of overall available services for cancer survivors, the most frequently referred to were allied health (80%), nurse-led care (80%), and advocacy or support groups (60%). Of those with defined eligibility criteria to access certain services for cancer survivors (67%), these generally pertained to location of residence, completion of curative-intent treatment including chemotherapy, meeting criteria based on patient-reported outcomes measure (PROM) scores, or tumour-specific clinic referrals.

**Table 2:** Summary of contextual survey data from virtual interviews, by centre

Variables	N (%)
Does your centre have dedicated CS services?	
Yes	6 (40)
No	9 (60)
If no dedicated CS services are available, where do you refer these patients to? (*n* = 9)	
Usual oncologist manages	9 (100)
Can you describe any CS services that you have available to refer to?*	
Allied health	12 (80)
GP-based care	6 (40)
Other specialist-led care	7 (47)
Nurse-led care	12 (80)
Advocacy or support groups	9 (60)
Classes or courses	6 (40)
Others	10 (67)
Unsure	2 (13)
Of the CS services named above, which ones are routinely referred to?*	
Allied health	6 (40)
GP-based care	2 (13)
Other specialist led care	2 (13)
Nurse led care	3 (20)
Advocacy or support groups	1 (7)
Classes or courses	2 (13)
Others/unsure	4 (26)
None	7 (47)
Eligibility criteria to access CS services?	
Yes No	10 (67)
	5 (33)
Do you have sufficient interpreter services available to meet your cancer care services' needs?	
Yes	8 (53)
No	7 (47)
Does your centre have any specific resources or supports for any patients affected by cancer from CALD backgrounds?	
Yes	10 (67)
No	4 (27)
Unsure	1 (7)
What resources, supports or services does your service provide? (*n* = 10)*	
Written	8 (80)
Support groups	5 (50)
Bilingual	2 (20)
Audiovisual	1 (10)
CALD officer	1 (10)
Electronic alerts	1 (10)
Are any of these specific to CS in patients from CALD backgrounds?	
Yes	3 (20)
No	12 (80)
Is your centre currently conducting any research focused specifically on CS programs or interventions in cancer survivors of CALD backgrounds?	
Yes	6 (40)
No	9 (60)

*indicates more than one response could be selected

Abbreviations: *CS* Cancer Survivorship; *CALD* Culturally and Linguistically Diverse; *GP* general practitioner

The median percentage of patients requiring language interpretation during consultations was 25% (*n* = 9, range 1–100%). Eight (53%) centres reported having sufficient formal interpreter services to meet their centres’ needs. Thirteen (59%) participants reported 15% (range 0–80%) of consultations using family members to interpret.

Ten (67%) centres reported having CALD-specific resources or supports for oncology patients, mostly as written resources (80%) or support groups (50%). CALD groups with in-language services available included Chinese, Arabic, Vietnamese and Macedonian. Three (20%) had CALD-specific, CS-specific services, including in-language survivorship written information for specific CALD groups, and CALD-specific CS support groups. Six (40%) centres had CALD-specific research activities, including GP shared-care programs, patient navigation and mentor programs, in-language resource/PROM development, and creation of CALD service directory.

### Qualitative data analysis

**Fig. 1:**
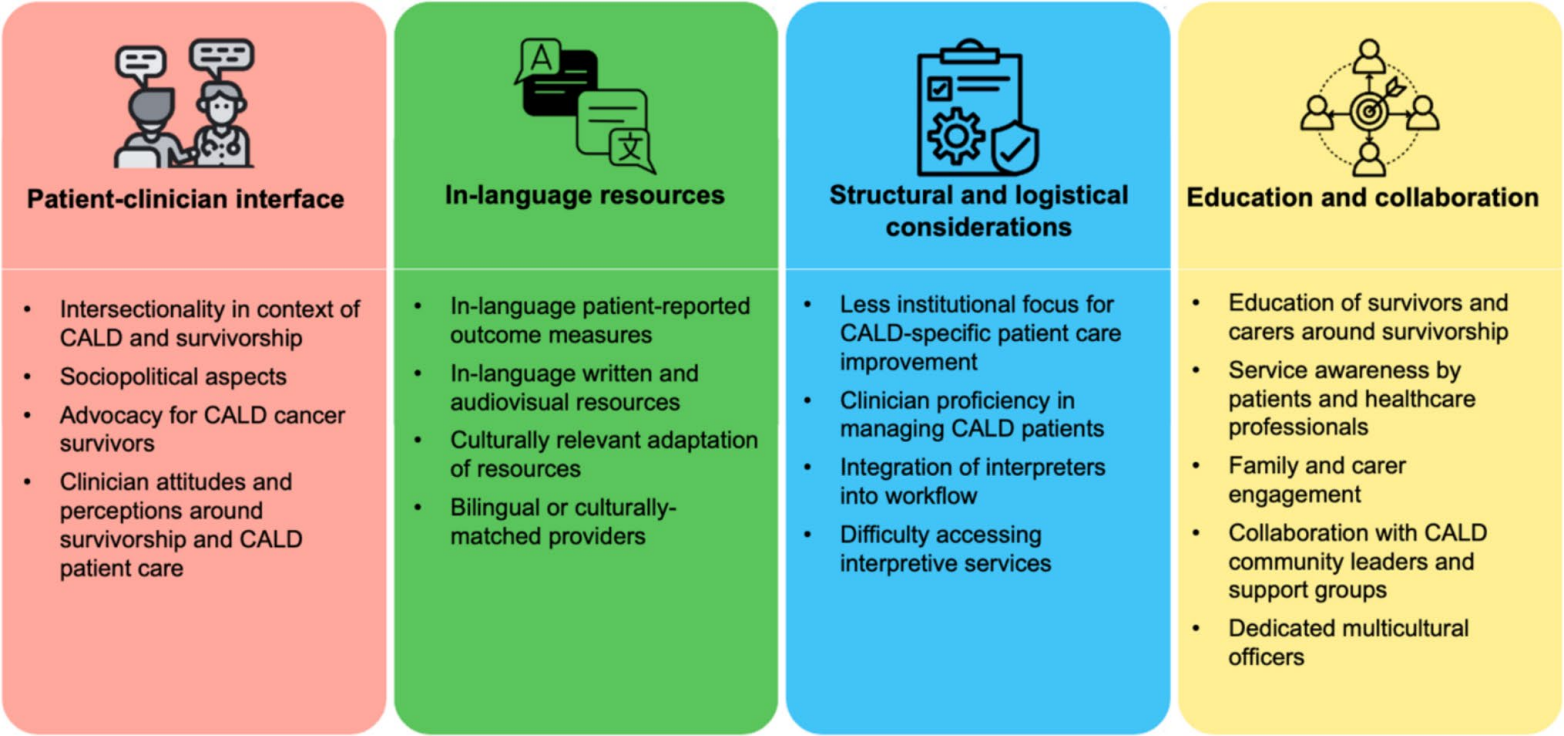
Summary of factors identified from qualitative analysis that impact care of cancer survivors from culturally and linguistically diverse backgrounds. Abbreviations: CALD = Culturally and Linguistically Diverse

Qualitative analysis identified four themes (Fig. 1 and Supplementary Table 1): Patient-clinician interface; In-language resources and staff; Structural and logistical considerations; and Education and collaboration.

### Theme 1: Patient-clinician interface

Participants reported that discussions between clinicians and patients were already complex and multifaceted within the realm of cancer survivorship, noting this was further intensified in CALD-specific survivorship care as exemplified below:

I have to refer to intersectionality on this one… it's that privilege, oppression, education… But I think the CALD are particularly marginalised in understanding cancer, how to interact and act upon a cancer diagnosis… when you look at culture, what you see is that tip of the iceberg. But when you go down beneath that water level, there is stuff there that obviously is not overt, but it is there. And that's what actually impacts the stuff that you don't see, it's what impacts our interaction with cancer, our understanding of cancer is those belief systems and that they're not to be minimised… they should never be reduced or ignored. (Participant 12)

Participants reported stigma associated with a cancer diagnosis in some individuals from CALD communities, which stems from core cultural or spiritual beliefs across different backgrounds. This can lead to minimisation of healthcare access following completion of primary treatment, or avoiding seeking help when necessary:

There's always this stigma that when you have cancer … ‘I'd rather not think about it. That's a path that I want to hide under the carpet. I don't want to talk about it. (Participant 18)

The lived experiences of Australian people with refugee backgrounds pose additional barriers to healthcare access, both in terms of health insurance status and psychological trauma associated with war and poverty. Whilst this is a challenge that can be delineated from CALD-specific survivorship issues, many refugees from CALD backgrounds face overlapping difficulties in healthcare access:

…overlying the issue of CALD population is an issue of refugee status. That's not equivalent to CALD but significant… they have unique issues relating to access to funding for services and access to privacy regarding their background. (Participant 5)

Another challenge is the approach from oncology professionals in providing survivorship care to CALD patients, particularly in context of their own belief systems and cultural aspects. Delivering tailored survivorship care for patients of CALD background requires patience and understanding, which is challenging due to time constraints, resource limitations, or issues with proficiency in managing CALD-specific issues:

…it's harder for CALD populations when you actually have to go through their cultures or their customs and try to get through accessing mental health… (Participant 20)

### Theme 2: In-language resources and staff

A key component of managing oncology patients from CALD backgrounds is ensuring communication factors are addressed. Lack of specific resources, or unavailability in specific languages, poses barriers to providing optimal care and addressing specific needs:

…when there just aren't resources available in a particular language that you're trying to provide support, and when you're trying to look at managing a specific symptom and you want to be able to give someone written information… it's a real barrier in providing care. (Participant 10)

Even with translated written resources, there may be issues with accuracy of translat0ion or cultural relevance for the group that the information is meant to support. This can lead to lack of engagement and reduced compliance with survivorship interventions or recommendations:

…if you translate something for language, it doesn't mean that it's actually culturally appropriate. So you might translate it word for word, but it means nothing because it's not talking about what's important to that culture. (Participant 4)

Participants reported an untapped resource is bilingual, culturally matched healthcare providers. Not only can they bypass the need for interpreters, but they can provide representation and reassurance from the perspective of cultural safety, mutual understanding and consequent increased engagement and uptake of survivorship resources:

…that's probably a big advantage to that, if you've got a GP or oncologist who speaks your own language, they may be more likely to find out about these sorts of resources. (Participant 1)

Understandably, placing additional responsibilities on staff from specific CALD backgrounds, particularly when numbers are limited:

…it's also a big responsibility… if we have one Vietnamese speaking nurse on duty, is it her responsibility to recruit all our Vietnamese-speaking patients? (Participant 14)

### Theme 3: Structural and logistical considerations

Participants reported caring for cancer survivors from CALD backgrounds increases the resources and time required at macro- and micro-organizational levels. This is exacerbated when there is limited or delayed access to formal interpretive services, or use of telephone or virtual interpreter services:

… the challenge of trying to get an interpreter for an important consultation is bad enough that when you're doing your follow-up simple consultations and talking about survivorship issues… often those translation services aren't accessed and therefore you're trying to give information in, circumstance that's not ideal and therefore you're giving truncated information. (Participant 6)

Integrating interpreters into clinical workflow is logistically challenging, requiring increased coordination, especially when booking interpreters for multiple patients or for individual patients with appointments with various healthcare professionals or services. In scenarios where interpreters are unavailable, family members are frequently utilised, but this reduces the ability to discuss more sensitive issues.

…when you're dealing with sensitive after-effects of treatment, translating via a younger male son about his mother, you know, survivorship doesn't really emerge as a key priority in those consultations. (Participant 13)

### Theme 4: Education and collaboration

Optimising CS care delivery for people from CALD backgrounds requires a collaborative approach between key stakeholders, including survivors, carers, oncology professionals and community leaders. The concept of CS and its importance is often misunderstood, which can impact engagement:

I think ‘survivorship’ as a term is such a nebulous one in English. I can't imagine how that translates to other languages. And trying to describe it is so difficult. (Participant 10)

Framing survivorship as a holistic approach to living well with, or after cancer can be crucial for conveying the importance of accessing available CS services:

…if we were able to educate patients that at least the service is available and include them and make sure that we do attend to referring to the clinic. (Participant 9)

In the absence of CS services, a directory of community-based services tailored to specific CALD groups could be a helpful resource:

…a central place where people could go for information, which refers you to the appropriate services.., but also obviously in different languages. (Participant 22)

A core component of CALD-specific survivorship services is collaboration with community leaders and support groups, which increases the likelihood that developments are tailored to the community’s needs:

…we try and have engagement as a hospital with communities and so to understand best how we can serve them. So we meet with the cultural leaders from various communities and tribes, and there's a group that is dedicated to that. (Participant 11)

## Discussion

This study characterised survivorship services specific to CALD populations in Australia, and explored cancer care professionals’ perceptions of factors influencing the delivery of survivorship care to cancer survivors from CALD backgrounds. While most centres had general cancer resources for specific CALD groups, only 20% offered CALD-specific survivorship services. Four key themes that characterised perceptions of CALD-specific survivorship care were highlighted including (1) the patient-clinician interface; (2) in-language resources; (3) structural and logistical considerations; and (4) education and collaboration. Our study findings expand upon the extant literature on CALD-based healthcare delivery, although several sub-themes specific to CS delivery were determined.

A key theme from this study’s findings pertains to the patient-clinician interface. Specific aspects for each individual CALD group affecting how they interact with the healthcare system include nuanced interplay between culture and language, and external socioeconomic, geographic and political factors. This concept is known as intersectionality, a theoretical framework outlining the intersection of various social categories within an individual reflecting privilege and discrimination [24, 25]. These factors have an additive impact on cultural beliefs and behaviours that CALD groups have pertaining to healthcare. In our study, these nuances were elicited in the context of caring for CALD cancer survivors, reinforcing the uniqueness of each CALD group and relevant social issues. However, we acknowledge that various intersectional issues, such as refugee status, are unrelated to culture and language, and require additional considerations.

As identified in our study, even within English-speaking communities, the term ‘Cancer Survivorship’ can be misunderstood. Whilst current definitions refer to cancer survivors as anyone diagnosed with or living with cancer, which also can be interpreted to include friends, family and caregivers as well as those with metastatic disease [26–28], the landmark 2006 Institute of Medicine report considered survivors those who had completed primary, curative-intent treatment [29]. Consequently, it is inevitable that translating these concepts into other languages can be misconstrued, though alternative terminology could be used to emphasise utility.

Our results emphasise the importance of recognising specific needs of each CALD group before implementing survivorship resources or supports. Currently, there is limited official guidance regarding how to develop culturally relevant materials for CALD groups, although this is an emerging priority area [30, 31]. Wiley et al. [9] reported on lessons learnt in developing written resources for cancer survivors of Arabic, Italian and Vietnamese descent and identified key tasks necessary in the process, including community engagement and consultation, and culturally sensitive content development. Our work reinforces their findings, particularly regarding co-design in written survivorship resources and development of survivorship services, models of care, and other resources. Engagement with community leaders and consumers are crucial to developing CALD-specific resources through co-design to ensure linguistic accuracy and cultural relevance.

This study identified that centres with available survivorship resources could be dichotomised into *clinic-based* or *needs-based* services, mostly implemented based on existing models of survivorship care [8, 32]. Utilisation of bilingual staff has been shown to facilitate care [16, 33], which is particularly salient for primary care physicians who bridge communication gaps and foster tailored healthcare experiences, to provide culturally appropriate management recommendations. However, these merits must be weighed against potential harms to staff, particularly those who have not received appropriate training to facilitate sensitive discussions. Interestingly, shared care models have been explored as an alternative to specialist-led survivorship care in non-CALD settings. However, results have been conflicting, likely due to variations in study design, interventions, technological factors such as electronic health records, and patient demographics [34]. Rather than utilising a blanket approach, conceptualisation and implementation of models of care should be tailored to unique needs of the health service, including the unmet needs of CALD groups in that region [35]. Shared care models for cancer survivors from CALD backgrounds are currently an underexplored research area.

Survivorship care delivery is complex and multifaceted, requiring input from various healthcare professionals and other support staff [8, 32, 35, 36]. One common theme underpinning many CALD-based projects is the involvement of professional interpreters [37–40], with many highlighting the interpreter’s role in communication accuracy, care satisfaction, and building rapport through cultural safety [33, 37]. Whilst bilingual survivorship interventions are feasible and beneficial for cancer survivors from CALD groups [16], significant logistical barriers exist in integrating interpreters into workflow. Previous literature indicates interpreters may perceive their role as advocates to ensure patients’ culture-specific needs are addressed [41]. Future collaboration with interpreters to implement effective, streamlined survivorship care interventions is paramount.

Based on our study, we believe research and infrastructure need to be prioritised to optimise care for cancer survivors from CALD backgrounds. A recent Delphi study identified numerous challenging, competing facets that contribute to delivering survivorship care which need prioritisation, yet accounting for CALD groups was only one sub-category [42]. It was encouraging in our study to see institutions conducting research aimed at improving survivorship care for CALD groups; however, stakeholder buy-in at healthcare institutions will be required to effectively implement changes, requiring structured pathways to access information, resources, and care needs, and expansion of research networks. This aligns with global efforts to improve diversity and inclusion in oncology, including clinical trial recruitment [43–45].

This study has several limitations. We focused exclusively on oncology professionals, which may not represent patient perspectives. Purposive sampling was skewed due to limited responses from initial contacts, potentially affecting generalisability. Data collection issues such as social desirability responses and recall bias, could affect data accuracy. Finally, some of our findings may be specific to Australia and its healthcare system.

## Conclusions

The population of cancer survivors continues to grow worldwide, particularly as advances in therapeutics lead to unprecedented improvements in survival rates for many cancer types. Survivorship care is a key component of maintaining patient wellness regardless of remission status; however, service delivery remains challenging. As migrant populations increase in Australia, the number of cancer survivors from CALD backgrounds will also rise. It is becoming increasingly important to address the gaps identified in this study in the delivery of survivorship care for these populations, particularly as only a minority of centres reported access to CALD-specific survivorship services. We make a call to action for oncology clinicians and relevant stakeholders: to deliver the best care for cancer survivors from CALD backgrounds, utilising appropriate services to bridge disparities and improve outcomes, and develop tailored survivorship services and models of care need using a collaborative approach to prioritise cultural relevance and health service engagement.

## Supplementary Information

Below is the link to the electronic supplementary material.

Supplementary file1 (DOCX 26 KB)

Supplementary file2 (PDF 500 KB)

Supplementary file3 (DOCX 22 KB)

## Data Availability

The datasets generated and analysed during the current study are available from the corresponding author upon reasonable request.
