# Supplementary material for: What services are available for culturally and linguistically diverse (CALD) patients in the cancer survivorship setting? An Australian study
Source: Support Care Cancer. 2025 Mar 21;33(4):309. doi: 10.1007/s00520-025-09348-2 (PMC11928404; doi:10.1007/s00520-025-09348-2)
Supplement: Supplementary file 1 — Supplementary file1 (DOCX 26 KB) [file 520_2025_9348_MOESM1_ESM.docx]

**Supplementary Table 1. Summary of main themes, subthemes and additional participant quotations around factors that influence access and engagement of CALD populations with cancer survivorship services.**

| **Subtheme** | **Participant number** | **Quotations** |
| --- | --- | --- |
| **Theme 1: Patient-clinician interface** | | |
| Understanding intersectionality in the context of CALD and cancer survivorship | 20 | *“Another barrier really is preconceived ideas depending on the countries that they originated from and the traditional beliefs that they carried over, although they've been living in Australia for a long time.”* |
|  | 4 | *“The thing about the Chinese culture is that if you have a symptom, then you're really sick. And if you don't have any symptoms, then nothing is wrong with you and you are well. So it's hard sometimes to provide advice to say, "this is what you need to do to stay well, even though you feel well, now it's important for you to do these, take your medication, get the checks" because they're like, "I feel fine now. So everything must be fine." But as soon as they get a symptom they are like, "I'm dying, doctor. I'm dying".”* |
|  | 2 | *“Survivorship is similar to clinical trials… an additional piece of icing on the cancer journey that can only be accessed by a few who have the time, resources, willingness, understanding, literacy to get there. So you have to be aware of that inequity… Otherwise survivorship just becomes another instrument of oppression where people who are poor just can't get it.”* |
| Sociopolitical aspects of CALD | 5 | *“Overlying the issue of CALD population is an issue of refugee status. That's not equivalent to CALD but significant, but some people of CALD background are actually refugees and they have unique issues relating to access to funding for services and access to privacy regarding their background. And that can be a barrier.”* |
| Advocacy for CALD cancer survivors | 1 | *“…Some of the interpreters are probably advocates and so I suspect that some of the interpreters might actually say, ‘Hey, did you know about this information, or did you know about this support service?’ I think that they probably act as a bit of a guide in signposting people to services and resources.”* |
|  | 8 | *“Our health navigators, they're not there to be a translator. They're there to offer emotional support and encourage a patient to ask questions from their treatment team.”* |
|  | 11 | *“I think having discourse with representatives from the communities or particularly the patients themselves is important and these things are translatable. So having patient advocates is a very big part of survivorship and thinking… that should also include patients from CALD backgrounds.”* |
| Clinician attitudes and perceptions around cancer survivorship and CALD patient care | 20 | *“It's harder for CALD populations when you actually have to go through their cultures or their customs and try to get through, accessing mental health or accessing psychology is okay. So I think a lot of clinicians will just say, “okay, that's fine. Whatever you want to do, whatever you're comfortable with,” and then they'll just move on and then move on clinic to the next patient.”* |
|  | 1 | *“If you're seeing a nurse who's very savvy to things, then they'll probably talk to you about a number of different services and they'll probably ask about, “do you have a need for these sorts of things?” Because they're aware of the services that would match that unmet need, but there'd be lots of people who'd go, “super busy, how are you doing? Okay, that's good. See you later.” …there's been no attempt to screen for a whole bunch of needs, nor is there an attempt to go to match that person to a range of services they might be aware of.”* |
|  | 2 | *“I talked to some oncologists and they are just not sold on survivorship. I think it's got an image problem and I think there's a need to either educate or change the thinking. I think survivorship is still a fringe and the only way you get traction is to make it mainstream. So I don't know, because that's the only way you get more traction. How do you focus? How do you convince a sceptic that this is something worth investing in?”* |
| **Theme 2: In-language resources and staff** | | |
| Bilingual or culturally matched providers | 8 | *“…The reason we've got these navigators and the people that we've chosen, have been chosen because of their cultural connection and because of the way that they fit in with the cultural group that we're talking to... So that they can allow patients to talk freely and encourage them to continue to query what's happening to them and what needs to happen next.”* |
|  | 4 | *“I think that using bilingual staff would be wonderful… It just takes away that language barrier immediately. And I think a lot of patients might be booking in to see me because they're secretly hoping I can speak Chinese, but I don't.”* |
|  | 19 | *“If service providers have the ability to provide workers from different cultural background and if they're able to provide the same worker to the families… many elderly [patients] have talked to me that, “I'm happy with the services provided by so-and-so person. But yeah, I have no assurance that that person will continue providing care for me.”* |
| In-language resource availability | 8 | *“If they're going to put together a video they've got to be thinking about doing it in multiple languages and doing them all at the same time sort of thing. But I don't think just shooting it once and then dubbing it over, I don't think that style works anymore. I think they've gotta be a little bit more discerning than that.”* |
|  | 2 | *“I know that the Caucasian privileged patients at [institution] are getting some fantastic care after their cancers. It's really fantastic for them to have good psychology and good integration and good etc. but a lot of our patients don't get that. And I think that inequity in distribution of the fruits of research is not fair. So finding accessible ways in a resource poor environment is really important.”* |
| In-language PROMs | 10 | *“As part of our service for everyone who's coming in, they complete a number of patient reported outcome measures. And we have those in both English and Chinese, so we've had those translated. So in terms of I guess the operation of our clinic, we have got some, what I guess is a standard sort of survey collected in two languages.”* |
|  | 8 | *“… I think 49% of our patients overall speak a language other than English at home. So we're quite a diverse [local health district]. And the PROMs is currently in eight languages.”* |
|  | 9 | *“…they get about 10 or 12 forms to fill in, so I think that would be difficult. So I haven't actually referred anybody that's non-English speaking at the moment as our criteria is open to everybody within our eligibility. But I think it would be challenging for somebody to do all of that, that requires an interpreter for both.”* |
|  | 14 | *“We used the EQ5D and the ESAS and a Supportive Care Needs Survey ahead of appointments. So pre-COVID we were giving it to patients on an iPad in the waiting room and then we went to delivering it by pushing a link via SMS to patients ahead of their appointment. The initial phase was all in English, and what we found was that the people who had a language other than English as their first language were disproportionately not participating.”* |
|  | 21 | *“When you look at the NCCN distress thermometer, the translations. They don't necessarily, if you actually give them to patients, they don't necessarily translate exactly, from what the patients tell me.”* |
| Primary care physician involvement in ongoing care | 4 | *“I think the GPs probably play a big role in this. So I know that people from CALD backgrounds often do rely on bilingual GPs… it's kind of a untapped goldmine, workforce that we're probably not really supporting enough.”* |
|  | 21 | *“…I always ask the patient …, "does your GP speak your language?" Because I would often go get them to go see their GP... because a GP can communicate directly with them. Yeah, and I think we don't utilise that relationship well enough.”* |
| **Theme 3: Structural and logistical considerations** | | |
| Less institutional focus for improvement of CALD-specific patient care | 1 | *“Some of the facilitators would be the same, whether they were CALD or not, which is organisational imperative and direction, and then a process and defining the process and defining what happens in the process and who does what, and then counting it.”* |
|  | 4 | *“I think we need to do more research on it. I think we need to understand what, what the barriers are from, from the consumers because I'm just kind of thinking of the barriers as a clinician, but I don't know, I don't have the lived experience… So I think we need to build this model of, you know, and need to be done with each culture because you can't take from one and then plonk it into the other.”* |
|  | 11 | *“If you're developing, let's say a cancer survivorship tool. It should just be routine that somebody is working on translating, providing a copy that's suitable for different cultures… it's sensible for different centres to work out what their highest populations of need are and concentrate on them… making it that your policy is that if you're developing a new patient tool that it should be routine that your most represented CALD groups are included and thinking about how that's distributed, how that's created, what it looks like, what language it's in.”* |
|  | 22 | *“I don't think there's been a huge push for it, unlike, say [institution] or [institution], which has a big population that needs that. So I think it's sort of been off the radar for a long time and hasn't really been looked at as a potential way of improving patient satisfaction in care.”* |
| Clinician difficulties managing CALD-specific issues in context of workload | 9 | *“In terms of cancer services, I think they're a group perhaps that needs a little bit of extra support with a few things… sometimes that's a difficult thing to cover when you need access to interpreters and you've got some cultural sensitivities sometimes that are difficult for us to balance along with everything else.”* |
|  | 11 | *“Some clinicians… I think that being honest there is some discomfort sometimes. And it's when you have a long running clinic and you've already spent half an hour talking about things, I think that there are time pressures that perhaps may mean that things might get rushed or missed.”* |
| Clinician proficiency in managing CALD patients | 20 | *“…we need to actually educate clinicians about communication… for the CALD population, I don't think that there would be any barrier at all if there was a program that you go onto. The only problem would be the interpreter service, because you can't go through a dietician consultation with your interaction being either this or that… for OTs and physios, many times there's this huge miscommunication and misunderstanding. And then they just, you know, they build their recommendations on that misunderstanding.”* |
| Access to interpretive services, regardless of format | 10 | *“There can often be issues with the interpreters themselves in terms of what they've translated and what the patient's heard. Often family members will know who the interpreter is, a part of their community, and they would not like that. Some patients don't want translation as well. Some communities don't want their loved one to know what's going on exactly.”* |
|  | 14 | *“…sometimes we use the family member because we can't get an interpreter. I've got one lady, for example, who speaks Hakka Timorese, and we can almost never get an interpreter for her. There's no in-house service and the external agency is very ad hoc. She brings the daughter with her every time to help.”* |
|  | 10 | *“There's also been the innovation of virtual video interpreters, so that has become something we're all getting more used to, getting the video interpreters in for our clinic, but that requires clinic set up, so a screen, a camera and clinicians trained to use it.”* |
| Integration of interpreters into workflow is challenging | 4 | *“…knowing if someone needs an interpreter is really important… system to flag that and ensuring that there's extra time for that person and an interpreter is booked… I'm looking at my list and going, ‘Oh, that's a foreign name. I better just check if they need an interpreter.’ So I'll go and look at their notes and go, ‘Oh yes, they need an interpreter,’ all these other things, because my [patient navigator] that doesn't necessarily flag it and then book it. There needs to be a system and then I need extra time.”* |
|  | 10 | *“I had a patient who required CT. There's no information on preparation of CTs in other languages. If they don't take the time to actually call a patient with an interpreter… trying to do it in English. It's not great. It just doesn't work.”* |
|  | 11 | *“…the simple things, like even organising appointments. It's one thing to explain to somebody who might be from an English-speaking background, “okay, so, you know, you're going to meet with an endocrinologist, you're going to meet with a lymphedema nurse, you're going to have a bone mineral density test.” …But then having to translate that across and explain again is a potential barrier…”* |
|  | 22 | *“We do have a flag in our system. So in [electronic medical record], we can flag that they might need an interpreter. So we have that that comes up for administrative staff and clinical staff”* |
|  | 2 | *“It's not a simple problem because delivering interpreters to the point they need it, is definitely quite complex… a busy outpatient schedule might have 15 or 20 patients… I need an interpreter at 2.40, but oncology clinics always run overtime... And interpreters don't wanna waste time either.”* |
|  | 19 | *“It's about the waiting time involved for all this as well for someone from the CALD community to say yes to services itself is difficult, and when they say yes to that, the process isn't that easy, especially through My Aged Care. They get a lot of phone calls. They have to provide consent for children to talk on their behalf. And yeah so to get an update, it's difficult for them to reach out to so many providers.”* |
| **Theme 4: Education and collaboration** | | |
| Education of survivors and carers about cancer survivorship, in context of cultural awareness | 10 | *“…if I frame it as surveillance, they're pretty happy because that's medical. But survivorship doesn't have the same kind of meaning for them… particularly for CALD, there's probably lack of understanding of what survivorship means and how that can support… it could be a misunderstanding of the word survivorship and the support available within the survivorship program.”* |
|  | 4 | *“They probably are not aware of survivorship care… that there is a thing called survivorship care. They do want to talk about how to stay well... "Okay, I want to do what I can to stay well", but they I don't think they have a word for survivorship.”* |
| Collaboration with CALD community leaders and support groups to build relationships | 12 | *“One of the things that we understand around CALD is connecting, visual connection, connecting with one's own community. That's very powerful. You would get further along when you connect if it's something that you're looking to do rather quickly, engaging with those from your community, particularly leaders. I think you have to go to church priests, community leaders, they are a powerful voice and they are very well respected in their communities.”* |
|  | 4 | *“[Support group] does have a presence in the centre, they provide volunteers. And I think at least once a year, they'll do a seminar which is very well attended. And then I get a big spike of referrals after that.”* |
| Service awareness by patients and healthcare professionals | 1 | *“Undoubtedly, patients don't know about stuff. And so, you know, I've never had a patient say, “Oh, do you have a survivorship booklet in Chinese?” No one has ever said that to me. Whereas, you know, I'll go, “Oh, I can finally give out one of those information booklets, and so I'll make sure I've got one and then I can refer people to, by the way, there's information on our website.””* |
|  | 21 | *“…if you've been to the GP and asked for mental health care plan, they give you a list of psychologists rather than being able to refer you to one particular psychologist. Let alone one that may be available to speak who speaks another language.”* |
|  | 14 | *“I might know what's immediately around [institution], some of them are driving an hour to come and see us at [institution], so I don't know what's in their area. So having some sort of network of where to find services near you. Let alone a directory that says where can I find, you know, Chinese speaking services near you?”* |
|  | 15 | *“Having a directory of community-based services… a central point that we could actually have a list of everything that's out there, that we could have better referral pathways. So I think knowing about services is the first step in being able to refer someone to a service… There's probably things that we don't even realise that communities are doing that we could potentially link more patients into.”* |
| Dedicated multicultural officers | 3 | “*Our multicultural team are really keen for information sheets to be in language as opposed to just a straight word-for-word translation.*” |
| Family and carer engagement | 6 | *“The main other thing will be engaging their family members and support systems in the community to try to help them to access these things as well, because that's obviously really important for some people from different cultural backgrounds to have the support of their family or for their family to think of it as an important aspect of their care.”* |

**Abbreviations:** CALD = Culturally and Linguistically Diverse; PROM = patient-reported outcome measures
