# Supplementary material for: What services are available for culturally and linguistically diverse (CALD) patients in the cancer survivorship setting? An Australian study
Source: Support Care Cancer. 2025 Mar 21;33(4):309. doi: 10.1007/s00520-025-09348-2 (PMC11928404; doi:10.1007/s00520-025-09348-2)
Supplement: Supplementary file 3 — Supplementary file3 (DOCX 22 KB) [file 520_2025_9348_MOESM3_ESM.docx]

**Appendix 2.** Survey questions developed by study team for interviews.

| **Survey interview Questions** | | | |
| --- | --- | --- | --- |
| # | Questions | ✓ | Answers |
| 1 | Does your centre have dedicated cancer survivorship services? |  | Yes (Go to question 3) |
|  |  |  | Unsure (Go to question 2) |
|  |  |  | No (Go to question 2) |
| 2 | Where or who do you refer these patients to? |  | No referral – usual oncologist manages (Go to Question 3) |
|  |  |  | Internal referral to another doctor eg GP / oncologist (Go to Question 3) |
|  |  |  | Other survivorship clinic – Where?  (Go to Question 3) |
|  |  |  | Others/unsure:  (Go to Question 6) |
| 3 | Can you **describe any survivorship services** that you have available to refer to (Prompt interviewee)? Within those services, are there established pathways?  (Examples:  Allied health – dietitian, exercise physiologist, physiotherapy, lymphoedema specialist, psychologist  Other specialist – cardio-oncology, geri-oncology, rehab, occupation physician) |  | Allied health |
|  |  |  | GP-based care |
|  |  |  | Other specialist-led care |
|  |  |  | Nurse-led care |
|  |  |  | Advocacy or support groups |
|  |  |  | Classes or courses |
|  |  |  | Others/unsure (please specify): |
| 4 | Of the services named in question 3, which services are survivors **routinely referred** to? (Prompt interviewee) |  | Allied health |
|  |  |  | GP based care |
|  |  |  | Other specialist-led care |
|  |  |  | Nurse-led care |
|  |  |  | Advocacy or support groups |
|  |  |  | Classes or courses |
|  |  |  | Others/unsure (please specify): |
| 5 | Are there any **specific eligibility criteria** to **access** certain survivorship services? For example, patients must have completed curative-intent treatment vs. inclusion of those with stable metastatic disease |  | No/Unsure |
|  |  |  | Yes – please expand: |
| 6 | You reported that __% of patients seen at your cancer care facility are from CALD backgrounds. What % of these patients would require interpreter services (in person, telephone or family)? |  | Answer: |
| 7 | Do you have sufficient interpreter services available to meet your cancer care services’ needs?  Approximately what proportion rely on or use family members to interpret during consultations? |  | Answer: |
| 8 | You reported that _______ groups were the most common CALD populations seen at your cancer care facility. Which groups require interpreter services most frequently?  Are there other CALD groups not mentioned above that also require interpreter services for a substantial proportion of consultations? |  | List of CALD groups |
| 9 | Does your centre have any specific **resources or supports for any patients** affected by cancer from **CALD backgrounds**? |  | Yes (Go to question 10) |
|  |  |  | Unsure (Go to question 11) |
|  |  |  | No (Go to question 11) |
| 10 | What resources, supports or services does your service provide (eg. Written, online)? Who administers them?  Are any of these specific to cancer survivorship in patients from CALD backgrounds? How does this service qualify as a CALD-specific service? Please elaborate. |  | List of resources, supports or services |
| 11 | Is your centre currently conducting any research focused specifically on cancer survivorship programs or interventions in cancer survivors of CALD backgrounds? If yes, can you describe the research? |  | Yes (Go to question 12) |
|  |  |  | Unsure (Go to question 13) |
|  |  |  | No (Go to question 13) |
| 12 | Are there any plans to integrate this research into routine practice? Please describe further: |  | Description |
| 13 | Is there anyone else in your service who I should be speaking to? |  | List of other potential interviewees |

Semi-structured questions about barriers and facilitators to quality, accessible survivorship care:

Now I’d like to finish off the interview by asking a couple of questions about barriers and facilitators to accessible, quality survivorship care in culturally and linguistically diverse populations.

14. You mentioned that ____ [services] are / are not available at your centre for cancer survivors of culturally and linguistically diverse backgrounds. What do you think are the main barriers to accessing survivorship care for your CALD patients?

15. What are the barriers that clinicians face in referring CALD patients to these services?

16. What do you think could facilitate survivorship service utilisation further for your CALD population group?

Eg. Education programs, clearer referral systems, involvement of other teams

17. Before we finish, are there any other issues specific to caring for cancer survivors of CALD backgrounds that you would like to comment on?
